# Clinical-Pathological Conference Series from the Medical University of Graz: Case No 170: A 33-year-old psychologist with severe dyspnea and right-sided chylothorax

**DOI:** 10.1007/s00508-020-01753-3

**Published:** 2020-10-29

**Authors:** Philipp K. Bauer, Martin Flicker, Elisabeth Fabian, Holger Flick, Luka Brcic, Bernadette Liegl-Atzwanger, Michael Janisch, Michael Fuchsjäger, Horst Olschewski, Guenter J. Krejs

**Affiliations:** 1 Division of Infectious Diseases and Tropical Medicine, Department of Internal Medicine I, Medical University of Vienna, Vienna, Austria; 2 Department of Internal Medicine, State Hospital Hochsteiermark, Leoben, Austria; 3 Division of Gastroenterology and Hepatology, Department of Internal Medicine III, Medical University of Vienna, Vienna, Austria; 4 Division of Pulmonology, Department of Internal Medicine, Medical University of Graz, Graz, Austria; 5 Diagnostic and Research Institute of Pathology, Medical University of Graz, Graz, Austria; 6 Division of General Radiology, Department of Radiology, Medical University of Graz, Graz, Austria; 7 Division of Gastroenterology and Hepatology, Department of Internal Medicine, Medical University of Graz, Auenbruggerplatz 15, 8036 Graz, Austria

**Keywords:** Cystic lung disease, Lymphangioleiomyomatosis, VEGF-D, Sirolimus

## Presentation of case

### Dr. H. Flick:

A 33-year-old psychologist complained of palpitations and the feeling of tightness in her chest, dyspnea on exertion (and sometimes even dyspnea at rest) since 1 week ago. She has been taking hormonal therapy (progesterone/lynestrenol) because of endometriosis for the last 2 years. Her pulmonologist referred her to the emergency room (ER) of Graz University Medical Center because of rapidly progressive dyspnea and suspected pulmonary embolism. A mass had been detected in the retroperitoneum and biopsied in a hospital in another state 2 months prior to admission. Results of the biopsy were not available. Physical examination on admission was unremarkable except for complete dullness over the right lung on percussion. Blood pressure was 120/80 mm Hg, heart rate (HR) 138 beats per minute (bpm), temperature 36.0 °C, oxygen saturation at ambient air 95%. Electrocardiogram showed sinus tachycardia (HR 117 bpm), S1Q3 type, and P waves and PQ interval were unremarkable. Transthoracic echocardiography revealed normal aortic, mitral and tricuspid valves. The pulmonic valve was not assessable, tricuspid annular plane systolic excursion was reduced at 0.8 cm. When moving the sonography sensor to the abdomen, no ascites was seen. Laboratory tests: leukocytes 15.36 × 10^9/L (normal: 4.4–11.3 × 10^9/L), hemoglobin 16.8 g/dL (normal: 12–15.3 g/dL), hematocrit 50.3% (normal: 35–45%), gamma-glutamyl transferase 63 U/L (normal: <38 U/L), C‑reactive protein (CRP) 7.5 mg/L (normal: <5.0 mg/dL), D‑dimer 2.18 mg/L (normal: <0.5 mg/L).

X-ray and computed tomography (CT) of the chest revealed a massive pleural effusion on the right side. Immediate paracentesis in the ER yielded 1 L of a white turbid effusion. Detailed analysis see Table 1.

**Table 1:** Laboratory analysis of the patient’s milky pleural effusion

Parameter		Patient data	Common results in chylothorax
Leukocytes	Total (per µL)	7377	>400
	Lymphocytes (%)	76	>50
Total protein (g/dL)		4.6	2–4
Lactate dehydrogenase (U/L)		185	70–200
Glucose (mg/dL)		96	80–130
Cholesterol (mg/dL)		143	<200
Triglycerides (mg/dL)		3959	>110^a^ (diagnostic criteria)
pH		7.37	7.35–7.50

^a^Or presence of chylomicrons

A diagnostic test was performed and specific treatment was initiated.

### Dr. M. Fuchsjäger:

**Fig. 1:**
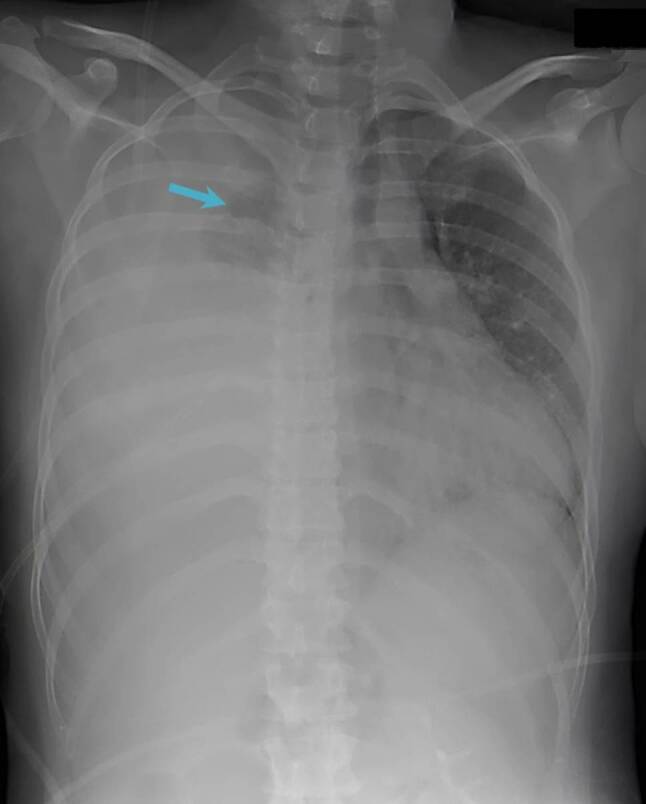
Chest X‑ray (in expiration) with subtotal opacity of the right lung and merely residual ventilation of the right upper lobe (*arrow*) as well as a mediastinal shift to the left

**Fig. 2:**
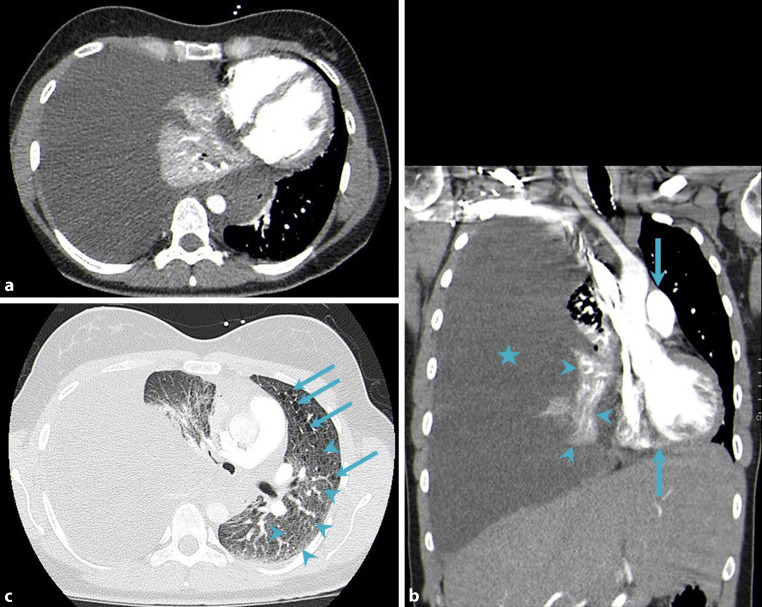
CT of the chest. Axial (**a**) and coronal (**b**) images (soft tissue window setting); massive right pleural effusion (*asterisk*), atelectasis of the right middle and lower lung lobes (*arrowheads*) as well as a marked mediastinal shift to the left (*arrows*). Axial image (**c**; lung window setting); multiple small lung cysts (diameter up to 6 mm; *arrows*) as well as extensive ground glass opacities (*arrowheads*)

The patient’s chest X‑ray showed subtotal opacity of the right lung with merely a residual ventilation of the right upper lobe and a mediastinal shift to the left (Fig. 1). Subsequent CT of the chest showed a massive right pleural effusion with subtotal atelectasis of the right lung and a marked mediastinal shift to the left. Multiple small lung cysts with a diameter of up to 6 mm as well as extensive ground glass opacities were seen in the left lung. There was no evidence of pulmonary embolism or enlargement of lymph nodes (Fig. 2).

## Differential diagnosis

### Dr. M. Flicker:

The patient under discussion is a 33-year-old woman who complained of progressive dyspnea, palpitations and chest tightness. Laboratory data revealed increased markers of inflammation and elevated D‑dimer. Chest X‑ray showed a large pleural effusion on the right; CT scan ruled out pulmonary embolism but revealed an opacity and multiple cysts in the left lung. The absence of ascites strongly suggests a problem of pulmonary or pleural etiology. Laboratory analysis of the milky pleural fluid revealed a right-sided chylothorax (i.e. accumulation of chyle within the pleural space). According to the appearance of a pleural effusion, different tests are indicated (Table 2) [1]. Chyle is described classically as having a white, milky or opalescent appearance [2]. A chylous effusion has a high concentration of triglycerides (>110 mg/dL), lymphocytes and immunoglobulins (Table 1) [3, 4]. If triglyceride levels are below 50 mg/dL, it is not a chylothorax. If a conclusive diagnosis cannot be made, lipoprotein analysis should be performed. The presence of chylomicrons establishes the diagnosis of a chylothorax [1]. Chylous pleural effusion or chylothorax occurs when flow in the thoracic duct, which carries fat after being absorbed in the intestine up to the superior vena cava, is compromised. It develops from disruption or obstruction of the thoracic duct or its tributaries with leakage of chyle from the ductal system into the pleural space. Chylothorax has various causes and is usually attributable to one of four etiologies [5]: (1) malignancy, (2) trauma (including surgery), (3) miscellaneous disorders and (4) idiopathic. Malignancy is the most common cause of chylothorax with lymphoma accounting for 37–75%, and bronchogenic carcinoma being the second most common reason [5, 6]. The proximity of the thoracic duct to the esophagus, aorta and spine, along with its variable anatomy, makes it particularly vulnerable during surgical procedures performed near these anatomical structures. Postoperative chylothorax has been described after nearly every known thoracic surgery as well as after neck surgery [7], but in rare cases may also occur after partial hepatectomy [8] (Table 3). Moreover, a wide variety of diseases, such as sarcoidosis, tuberous sclerosis, different infections or various lymphatic disorders have been associated with chylous pleural effusion (Table 3) [9]. Although the majority of causes for chylothorax listed in Table 3 can be excluded as a diagnosis in the discussed patient because of a negative history or lacking clinical features, it should be kept in mind that the patient had been diagnosed with a mass in the retroperitoneum 2 months before admission. However, since (1) laboratory data did not suggest lymphoma or leukemia, (2) there were no signs or symptoms indicating another malignancy (such as bronchogenic cancer) and (3) the described mass was anatomically not located in the proximity of the thoracic duct and, therefore, cannot be responsible for the development of the chylothorax, a malignancy seems very unlikely as the underlying cause of the chylous pleural effusion in this patient. Indeed, in this case the described mass is probably related to the patient’s endometriosis. Although the discussed patient had an increased level of D‑dimer, and subclavian venous thrombosis could basically lead to chylous pleural effusion, this diagnosis can also be ruled out because no thrombosis of the chest vessels was found on the CT scan.

**Table 2:** Tests indicated based on the appearance of the pleural effusion [1]

Appearance of fluid	Test indicated	Interpretation
Cloudy or turbid Turbid supernatant	Centrifugation Triglyceride levels	Turbid supernatant → high lipid levels >110 mg/dL → chylothorax >50 mg/dL but ≤110 mg/dL → obtain lipoprotein analysis Presence of chylomicrons → chylothorax ≤50 mg/dL and cholesterol >250 mg/dL → pseudochylothorax
Bloody	Hematocrit	<1% → non-significant 1–20% → cancer, pulmonary embolism, trauma >50% of peripheral hematocrit: hemothorax
Putrid odor	Stain and culture	Infection

**Table 3:** Etiologies of chylothorax [5–9]

Malignancy	Lymphoma, chronic lymphocytic leukemia Lung cancer Metastatic disease
Traumatic	Surgery for congenital heart disease, cardiovascular surgery (e.g. aortic procedures, coronary artery bypass graft surgery, valve replacement), esophagectomy, resection for lung cancer, lung transplantation, resection of mediastinal mass (e.g. neuronal tumor, thymoma, neuroblastoma, cystic hygroma, ganglioneuroma, metastatic tonsillar carcinoma), mediastinoscopy and lymphadenectomy, spinal surgery, central line placement, pacemaker implantation (via subclavian vein), embolization procedure for pulmonary arteriovenous malformation, partial hepatectomy, blunt chest trauma and other external injury
Miscellaneous disorders	Sarcoidosis, tuberous sclerosis, pulmonary lymphangioleiomyomatosis, congestive heart failure, Kaposi sarcoma, radiation therapy of the mediastinum, (Granulomatous) Infections: tuberculosis, histoplasmosis, filariasis Subclavian venous thrombosis Congenital or acquired lymphatic disorders: Gorham’s disease, Milroy disease, congenital lymphatic hypoplasia Various lymphatic diseases: lymphangiomatosis, yellow nail syndrome, lymphangioma, thoracic duct cyst Secondary to chylous ascites (due to e.g. alcoholic or cryptogenic cirrhosis, hepatitis C infection, primary biliary cholangitis, primary sclerosing cholangitis, cholangiocarcinoma, pancreatic carcinoma)
Idiopathic	Congenital (neonatal) Cases with no clear cause

Besides chylothorax, the finding of multiple lung cysts is also a key clinical feature in this patient, and various differential diagnoses should be included. Lung cysts may be congenital or occur due to Langerhans histiocytosis, rare genetic disorders, such as the Birt-Hogg-Dubé syndrome or various interstitial lung diseases [10]. Moreover, lymphangioleiomyomatosis (LAM), a multisystemic disorder characterized by the proliferation of smooth muscle cells resulting in cystic lung disease [11], should be considered in this patient. This disease is a rare progressive disorder with a prevalence of about 2.6 patients per 1,000,000 population [12]. It typically affects women of childbearing age with an estimated prevalence of 5 per 1,000,000 women [13]. Two forms of LAM present clinically—the sporadic LAM and tuberous sclerosis complex-associated LAM [14]. Pulmonary manifestations include multiple thin-walled cysts in the lungs and a high frequency and recurrence rate of pneumothorax (66%) and chylothorax (25%). As the disease progresses it leads to narrowing and obstruction of the airways and presents similarly to obstructive lung disease. It results in alveolar damage and the development of cystic disease of the lungs and the lymphatic system [11]. LAM patients lose about 90 mL of their forced expiratory volume in 1 s (FEV1) annually. Diffusing capacity for carbon monoxide (DLCO) is another measurement for disease progression and may be reduced even if FEV1 is normal. Both FEV1 and DLCO are related to disease progression [15]. Extrapulmonary features include angiomyolipomas and lymphangioleiomyomas in 20% of affected patients [16]. Vascular endothelial growth factor‑D (VEGF-D) is a biomarker of LAM and reflects disease severity [17]. Clinical observations found that the menstrual cycle influences symptoms and occurrence of pneumothorax. The decline in lung function slows after menopause and accelerates with exogenous estrogen use and pregnancy [18, 19]. Numerous studies have determined the role of estrogen and progesterone in the pathogenesis of LAM [20]. LAM lesions further share characteristics with uterine leiomyomas with both containing abnormal immature smooth muscle-like cells and expression of estrogen and progesterone receptors [21]. Concluding from animal models, the uterus may be the origin of LAM [22, 23]. Since LAM is caused by *TSC2* or *TSC1* mutations that result in overactivation of the mechanistic target of rapamycin pathway (mTOR), sirolimus (an mTOR inhibitor) has been approved for the treatment of LAM in many countries [14]. The effectiveness of sirolimus includes improvement of lung function, exercise capacity, oxygen level, quality of life and a decrease in VEGF‑D levels [24].

In summary, the following four key findings strongly suggest the diagnosis of LAM in this patient: (1) hormone therapy for endometriosis for 2 years, (2) a mass in the retroperitoneum, (3) chylothorax and (4) multiple cysts in the lungs.

In order to establish the final diagnosis, serum levels of VEGF‑D should be measured and the biopsy results of the retroperitoneal mass should be obtained.

An elevated D‑dimer is not associated with LAM. In this patient, pulmonary embolism was excluded by a CT scan; nevertheless, elevated D‑dimer levels may result from a thrombosis localized elsewhere in the body (e.g. deep venous thrombosis in the lower limbs) and should thus be searched for.

## Dr. M. Flicker’s diagnosis

Lymphangioleiomyomatosis

## Discussion of case

### Dr. H. Flick:

LAM is a rare cystic lung disease that was suspected by the physicians taking care of this patient. To confirm the diagnosis, serum levels of VEGF‑D were analyzed and results of the biopsy of the retroperitoneal mass were obtained as suggested by Dr. Flicker. VEGF‑D is a diagnostic biomarker, and an elevated serum level (≥800 pg/mL) can help confirm the diagnosis of LAM in women with a compatible medical history and characteristic CT scan of the chest [16]. VEGF‑D acts as a lymphangiogenic growth factor and may reflect the burden of LAM cells in the human body. LAM cells produce VEGF‑C and VEGF‑D which are involved in cell proliferation and lymphangiogenesis via VEGFR3; however, only VEGF‑D is elevated in serum [25]. In this patient, serum VEGF‑D was very high at 2623 pg/mL.

### Dr. L. Brcic:

**Fig. 3:**
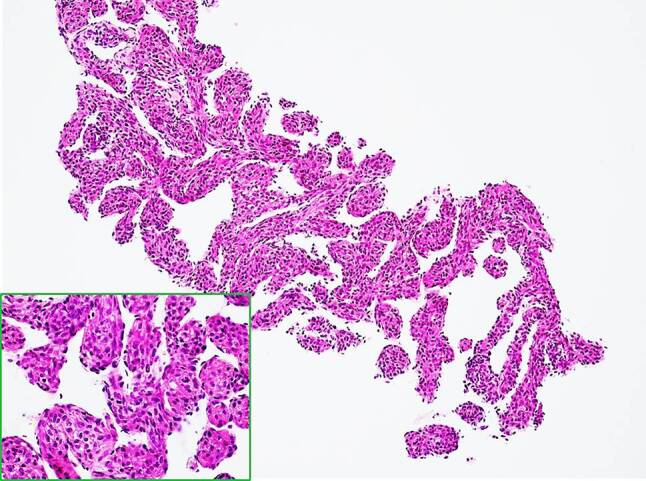
Histological presentation of the retroperitoneal mass. Spindle tumor cells without marked atypia are present, displaying eosinophilic cytoplasm and interspersed blood vessel structures (hematoxylin-eosin staining, objective ×4 and ×20 *inlet*)

Histology of the retroperitoneal mass biopsied 2 months before admission in a hospital in another state showed mesenchymal tumor tissue composed of spindle cells intermixed with blood vessels (Fig. 3). Tumor cells demonstrated minimal atypia and 1 mitosis per 10 high power fields (HPF) was detected. In immunohistochemical analysis, tumor cells were positive for human melanoma black 45 (HMB45) and microphthalmia transcription factor (MITF), and some cells even for α‑smooth muscle actin (αSMA), caldesmon and desmin. Staining was negative for pan-cytokeratin, epithelial membrane antigen (EMA), melan A, S100 and murine double minute 2 (MDM2). These results are consistent with lymphangioleiomyomatosis.

Lung biopsy is not required for the definite diagnosis of LAM [16] and will only be performed if the diagnosis cannot be made non-invasively. Histological and pathological features are proliferation of neoplastic smooth muscle cells, i.e. LAM cells which may be sparse in early stage disease. Immunohistochemical staining with αSMA, HMB45, estrogen receptor and progesterone receptor may be required to identify them. LAM cells consist of two types of cell subpopulations, fibromyoblast-like spindle-shaped cells which express smooth muscle specific proteins (e.g. α‑actin, desmin, vimentin) and epithelioid-like cells which express glycoprotein gp100, a marker of melanoma cells and immature melanocytes [14].

### Dr. H. Flick:

**Fig. 4:**
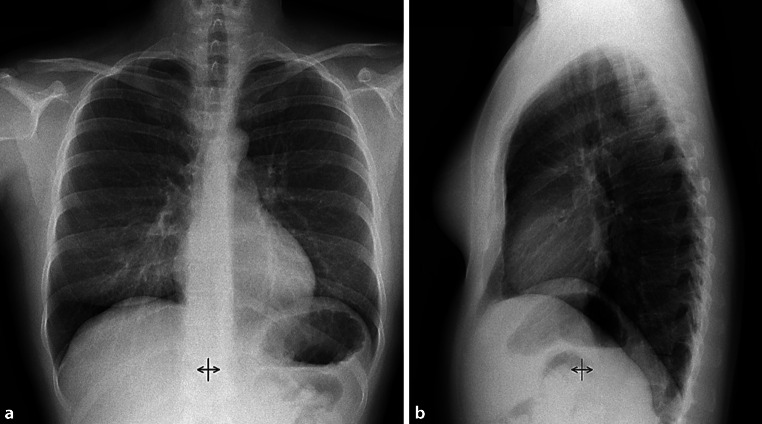
Normal follow-up chest X‑ray **a**; p.a. and **b**; lateral) after treatment with sirolimus for 6 months

After the diagnosis of LAM had been established, treatment with sirolimus was initiated with a dose of 4 mg per day. After 1 month of therapy and repetitive drainage of a total of 17 L of chylous pleural effusion, the patient’s condition stabilized and there was no recurrence of chylothorax (Fig. 4). During this treatment, VEGF‑D decreased from 2623 pg/mL to 595 pg/mL and the retroperitoneal mass decreased in size and was radiologically no longer detectable at 6 months follow-up. Dosage adjustment was carried out to achieve a serum drug concentration between 5 and 10 ng/mL [14]. During therapy with sirolimus, no relevant obstruction or restriction was found in the pulmonary function test (forced vital capacity, FVC: 128.5 L, FEV1% FVC: 75.5%); however, DLCO remained impaired (5.41 mmol/min/kPa corresponding to 60% of predicted) even on therapy, reflecting the damage that has occurred in the lung parenchyma, presenting as multiple lung cysts. Formation of lung cysts in LAM is complex: Mutation and loss of function of the *TSC* genes in LAM precursor cells leads to activation of mTOR signaling and the clonal expansion of LAM cells expressing smooth muscle proteins and markers of melanogenesis. In early stage disease these cells are sparse but form nodules in more advanced disease. These contain fibroblasts and lymphatic endothelial cells which are recruited or perhaps transdifferentiate under stimulation by lymphangiogenic growth factors, particularly VEGF‑D [26]. Inflammatory cells including T cells and mast cells are recruited and generate a mature LAM nodule [27]. Lung cyst formation may then occur by unregulated protease activity with the cysteine protease cathepsin K expressed in LAM nodules [28–30] and ineffective lymphatic remodeling [16].

LAM is a rare, systemic disease that is associated with cystic lung destruction, chylous fluid accumulation and abdominal tumors, including angiomyolipomas and lymphangioleiomyomas. Untreated LAM has a median survival of about 20 years after diagnosis [15]. In Austria, about 80 women carry the diagnosis of LAM; 12 of them are currently treated at the clinic for rare lung diseases of the Graz University Medical Center.

### Dr. G.J. Krejs:

For rare diseases, therapeutic options are often limited. Besides sirolimus, are there any other therapeutic options for patients with LAM?

### Dr. H. Flick:

Although there is evidence of estrogen involvement in the pathogenesis, the evidence for hormonal therapy in LAM is very limited and inconsistent. For example, a prospective study in premenopausal women with LAM did not find a beneficial effect of triptorelin (a gonadotropin receptor hormone analogue) on lung function during a 3-year observation period [31]. Data of another study in which letrozole (an aromatase inhibitor) was investigated in postmenopausal LAM patients were inconclusive, but suggested a potential beneficial effect on lung function and VEGF‑D levels [32]. The current American Thoracic Society/Japanese Respiratory Society (ATS/JRS) guidelines do not recommend using hormonal therapy, including progesterone, gonadotropin receptor hormone agonists, tamoxifen, aromatase inhibitors or oophorectomy [16].

Since levels of matrix metalloproteinases (MMP)-2 and MMP‑9 have been found to be increased in patients with LAM in serum and lung biopsies [33, 34], doxycycline, which inhibits the production and activity of several MMPs, has also been investigated as a therapeutic agent; however, a randomized, double-blind, placebo-controlled trial revealed no differences between doxycycline and placebo in the rate of FEV1 decline and quality of life [35]. Therefore, doxycycline is not recommended as a therapeutic approach in LAM [16].

Experimental studies further suggest a benefit of statins (atorvastatin and simvastatin) in LAM [36–38], but additional studies are required to investigate the effect in humans. Moreover, autophagy inhibitors such as chloroquine and hydroxychloroquine have been proposed as potential therapeutic agents in LAM. In vivo and in vitro studies found decreased TSC2 null cell survival and reduction in tumor size by chloroquine. This effect increased in combination with sirolimus [39]. The combination with hydroxychloroquine and sirolimus revealed improved lung function at 24 weeks, but lung function decreased at the 48-week time point [40].

In patients with severe LAM and hypoxemia due to a decrease in DLCO and/or FEV1, long-term oxygen therapy based on the experience in patients with chronic obstructive lung disease is recommended [41]. Long-term oxygen is particularly helpful in patients with PaO_2_ ≤ 55 mm Hg or SpO_2_ ≤ 88%, or in those with PaO_2_ ≤ 60 mm Hg if peripheral edema, polycythemia or pulmonary hypertension are present [14]. Patients with FEV1 below 40% of predicted are considered candidates for lung transplantation [42].

Patients with LAM are encouraged to maintain a healthy lifestyle, exercise regularly and avoid cigarette smoking. A balanced diet is recommended. In patients with chylothorax, a low-fat diet may be advised [14].

### Dr. G.J. Krejs:

LAM is a rare disease; in our 33-year history of holding clinical-pathological conferences at the Medical University of Graz, we had only one other case discussed in 1988 by Dr. Otto Brändli from Zurich, Switzerland. Sirolimus was not available back then. Although the disease is rare, interesting case reports are regularly published [43]. The disease presents with pulmonary features including dyspnea, pneumothorax, chylothorax and multiple thin-walled cysts in the lungs as well as extrapulmonary manifestations, such as abdominal angiomyolipomas and lymphangioleiomyomas. Elevated D‑dimer levels in this case are not a typical finding in LAM and are commented on by Dr. Olschewski.

### Dr. H. Olschewski:

Although the finding of increased D‑dimer levels may hint at the presence of a thrombus somewhere in the body, we should keep in mind that up to 70% of laboratory results with this biomarker in clinical routine are false positives in older patients. In the discussed (young) patient, the increased D-dimer levels may be due to the mechanical stress of vessels in the chest caused by chylothorax and a mediastinal shift. However, this parameter may also be a useful indicator for other conditions since data of the Lipid Study recently identified D‑dimer as a global predictor of mortality, i.e. not only cardiovascular, but also cancer-associated and all-cause mortality [44].

## Final diagnosis

Lymphangioleiomyomatosis
